# Improved Accuracy With a Novel Imageless Robotic-Assisted System Versus Manual Instrumentation in Unicondylar Knee Arthroplasty: A Cadaveric Study

**DOI:** 10.1016/j.artd.2026.102118

**Published:** 2026-08-24

**Authors:** Gary W. Doan, R. Patrick Courtis, Ian J. Leslie, Ugo Ihekweazu, David F. Dalury

**Affiliations:** a Research & Development, DePuy Synthes, Boston, MA, USA; b Clinical Research, DePuy Synthes, Leeds, UK; c Fondren Orthopedic Group, Texas Orthopedic Hospital, Houston, TX, USA; d Orthopaedic Institute, University of Maryland Saint Joseph Medical Center, Towson, MD, USA

**Keywords:** Unicondylar knee arthroplasty, Robotic-assisted, Manual, Alignment, Accuracy

## Abstract

**Background:**

Unicondylar knee arthroplasty (UKA) is a highly effective procedure for patients with end-stage single compartment osteoarthritis resulting in faster recovery and improved patient outcomes than total knee arthroplasty. However, higher revision rates remain a concern, contributing to increased utilization of robotic-assisted systems to support more accurate implant placement with the goal of reducing the most common reasons for revision.

**Methods:**

This study aimed to compare the resection accuracy of a robotic-assisted unicondylar knee arthroplasty (rUKA) compared to manual instrumentation (mUKA) in a controlled cadaveric study. Six surgeons performed bilateral UKA on 40 cadaveric specimens; rUKA on 1 leg and mUKA on the other. The specimens were computed tomography scanned preprocedure and postprocedure with the achieved resection angles compared to the planned. Resection depth accuracy was assessed by measuring the resected bone (with a caliper) accounting for the saw blade thickness.

**Results:**

The robotic-assisted UKA was found to have significantly greater accuracy for the angle of the resections than mUKA including tibial varus/valgus, tibial posterior slope, femoral varus/valgus, flexion/extension, and internal/external rotation. Both mUKA and rUKA procedures were found to have a high level of accuracy for the hip-knee-ankle angle and bone resection depths. rUKA had significantly fewer outliers than mUKA for the femur flexion/extension, internal/external rotation, tibial varus/valgus, and tibial flexion/extension.

**Conclusions:**

The rUKA demonstrated increased accuracy of the angle of resections compared to mUKA which may support increased consistency and reproducibility of the UKA procedure.

## Introduction

Unicondylar knee arthroplasty (UKA) is an effective procedure for patients affected by end-stage single compartment osteoarthritis affecting the knee. UKA is a less invasive procedure aimed at preserving more of the native joint compared to TKA with reduced bone resection and preservation of more of the patient’s native bone and soft tissues. In appropriately selected patients, this can result in fewer complications, better restoration of kinematics and improved patient outcomes compared to TKA [[1], [2], [3], [4]]. However, UKA remains underutilized due to UKA being associated with increased revision rates compared to TKA as well as the perception that it is a more difficult surgery compared to TKA. At 10 years, the overall revision rates for cemented UKA were approximately 3 times higher than cemented TKA with the most common reasons for revision being progressive arthritis, aseptic loosening, pain, implant wear, and instability [5].

To address this survivorship discrepancy, there have been improvements in both implant design and technique. There also has been an increased adoption of new technologies such as computer navigation and robotic-assisted systems [6]. These technologies may improve implant survivorship by enabling more accurate execution of the surgical plan as implant malposition is associated with an increased risk of revision [7,8]. The use of robotic assistance in UKA has been steadily increasing since 2014 with the proportion of robotic-assisted UKA (rUKA) procedures increasing from 9% to 53.4% in the Australian joint registry in 2024 [6].

**Figure 1:**
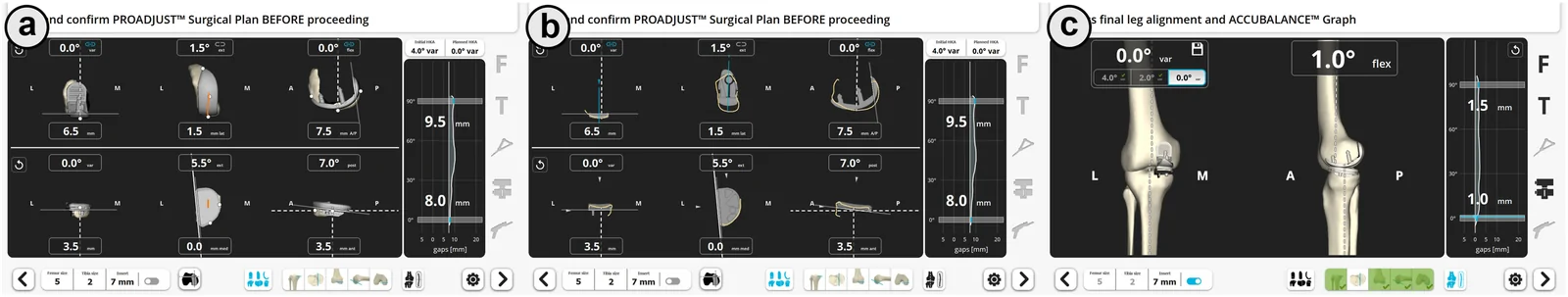
Planning screen (a), slice view (b), and final HKA assessment (c) from the VELYS Robotic-Assisted Solution UKA software application.

Multiple studies have shown improved resection accuracy of rUKA compared to manual instruments for semiactive systems such as NAVIO/CORI (Smith and Nephew, Pittsburgh, PA) and MAKO (Stryker, Fort Lauderdale, FL) [[9], [10], [11]]. This study aimed to compare the resection accuracy of the VELYS robotic-assisted solution (DePuy Synthes, Warsaw IN) system (Fig. 1) for UKA to standard manual instruments.

The VELYS RAS is an imageless system that directly controls the plane of resection of a handheld saw and facilitates the implantation of the fixed bearing Sigma HP Partial Knee system (DePuy Synthes, Warsaw IN). The VELYS RAS system was first released for use in TKA in 2021 but has subsequently been updated with new software and a narrower saw blade specifically designed for UKA procedures. Some new features introduced to the VELYS RAS UKA software include three-dimensional mapping of the bearing surface allowing slice view during planning and contact point tracking. The ability to generate a three-dimensional plan for a UKA procedure intraoperatively without the need for preoperative CT scan has the potential to reduce radiation exposure and reduce costs [12]. To our knowledge, this is the first study to report on the performance of the UKA application of the VELYS RAS.

## Material and methods

Six surgeons, with varied robotic/navigation experience and no prior experience with the VELYS UKA application (Table 1), performed bilateral medial or lateral UKA on 40 matched pair hip-to-toe cadaveric specimens (age: 67.53 ± 7.34 years [range: 49-80 years]; height: 66.38 ± 3.2 in [range: 61-72 in]; weight: 121.78 ± 28.83 lbs [range: 80-184 lbs]; body mass index: 19.45 ± 4.56 [range: 13.29-30.91]). Each surgeon performed rUKA with the VELYS RAS UKA on a randomized side and performed manual UKA (mUKA) with the SIGMA HP Partial Knee System with INTUITION Instrumentation (DePuy Synthes, Warsaw, IN) on the contralateral side. Each surgeon underwent standardized sawbones-based training with both the robotic-assisted device and manual instruments prior to performing their first case.

**Table 1:** Summary of experience level of surgeons involved in the execution of the procedures.

Surgeon	UKA volume (medial + lateral) per year	UKA volume (lateral only) per year	Current technique	VELYS TKA experience?
Surgeon 1	Mid (50-99)	None	Manual w/ Sigma HP	Yes
Surgeon 2	Mid (50-99)	Mid (˃10)	Robotic w/ MAKO UKA	No
Surgeon 3	Low (between 10 and 50)	None	Manual w/ ZB	No
Surgeon 4	Low (between 10 and 50)	None	Manual w/ Sigma HP	No
Surgeon 5	High (over 100)	None	Manual w/ Sigma HP, Robotic w/ NAVIO/CORI UKA	Yes
Surgeon 6	Low (between 10 and 50)	None	Manual w/ Sigma HP	Yes

### Robotic-assisted surgical technique

For rUKA cases, optical reference arrays were rigidly fixed to the distal femur and proximal tibia using Schanz screws. The specimen’s native anatomy was registered which involves the acquisition of key anatomic landmarks, the articular surface, and the specimen’s native kinematics through an entire knee range of motion. After registration, an intraoperative plan was generated (and stored in the system) with the goal of resurfacing the degenerative articular surface. The robotic-assisted device aligns the end effector, equipped with a motorized instrument and saw blade, to the planned resection planes where the surgeon can perform the tibial and femoral resections. Standard retractors were used to protect the surrounding soft tissue structures. A manual reciprocating saw was used when needed. After the primary resections, the femoral finishing block was navigated and pinned to facilitate femoral finishing. Once trial implants were inserted, the knee’s soft tissue balance, component contact tracking through a range of motion, and the final hip-knee-ankle (HKA) angle were evaluated.

### Manual instrument surgical technique

Optical reference arrays were also fixed to the distal femur and proximal tibia in mUKA cases to collect data such as preoperative and postoperative HKA. The surgeons were blinded to any information displayed by the system during the procedure and the optical reference arrays were positioned to avoid interference with the manual instrumentation during the procedure. Similar to the robotic-assisted surgical technique, surgeons registered the specimen’s native anatomy using the robotic-assisted system. After similar landmark registration, the procedure was completed with only manual instruments using the Sigma HP Intuition instruments per the surgical technique and the knee was trialed and evaluated as per the rUKA cases. Surgeons noted their surgical plan intraoperatively which was recorded manually.

### Resection angle measurement

For each specimen, a preoperative computed tomography (CT) scan was taken, with a 0.6-mm slice thickness and interslice distance. After the UKA procedures, the trial implants were carefully removed and the specimens were postoperatively CT scanned to capture the resection surfaces. The preoperative and postoperative CT scans were segmented and registered together in GeoMagics (3D Systems; Rockhill, SC) by operators who were blinded to cohort allocation. The femur and tibia anatomic coordinate systems were constructed from anatomic landmarks, identified on preoperative CT scans, and knee kinematics from intraoperative measurements. The superior-inferior (S-I) axis of the femoral coordinate system was aligned to the femoral mechanical axis (defined as a line from the femoral intercondylar notch to the geometric center of the femoral head). A temporary anterior-posterior (A-P) axis was defined as the femoral centerline (a line created by marking the center of the femoral condyle in extension and in flexion). The medial-lateral (M-L) axis was defined as orthogonal to the S-I and temporary A-P axes. Finally, the A-P axis was redefined to be orthogonal to the S-I and M-L axes, with the origin defined at the femoral intercondylar notch. Similarly, the S-I axis of the tibial coordinate system was aligned to the tibia mechanical axis (defined as a line from the center of the malleoli to the posterior aspect of the anterior cruciate ligament). A temporary A-P axis was defined from the posterior aspect of the anterior cruciate ligament to the medial third of the tibial tubercle. The tibial M-L axis was defined as orthogonal to the S-I and temporary A-P axes, and the A-P axis was redefined to be orthogonal to the S-I and M-L axes. For both femur and tibia coordinate systems, once axes were defined, the transverse plane was defined as normal to the S-I axis, the sagittal plane was defined as normal to the M-L axis, and coronal plane was defined as normal to the A-P axis. For each resection, the resected surface geometry was isolated, fit with a plane, and measured by a single blinded operator.

The femur varus-valgus (V-V) angle was defined as the angle between the distal femoral resection and the S-I axis in the coronal plane. The femur flexion-extension (F-E) was defined as the angle between the femoral posterior resection and the S-I axis in the sagittal plane. The femur internal-external (I-E) was defined as the angle between the posterior resection and the A-P axis in the transverse plane. The tibia V-V angle was defined as the angle between the tibial resection and the S-I axis in the coronal plane. The tibia A-P slope was defined as the angle between the tibial resection and the S-I axis in the sagittal plane.

### Resection depth measurement

The resection depths of the distal femoral, posterior femoral, and proximal tibial resections were measured intraoperatively for all specimens. After the primary resections, the bone remnants were measured using digital calipers (Mitutoyo 500-196-30) and compared to the surgical plan. The bone remnant was placed on top of the saw blade’s surface (which has a thickness of 2 mm) to measure the bone remnant and kerf of saw blade together. The fragment-saw construct was measured from the blade’s bottom surface to the highest point on the articular surface or to a point marked with a surgical felt tip pen to define the surgeon referenced point. During study initiation, an interoperator assessment was performed, in which 2 trained operators independently acquired a single measurement on the same specimen using digital calipers. The measurements were reviewed by a blinded engineer and demonstrated low interoperator variation, indicating comparable measurement performance between operators. Based on this assessment, all subsequent measurements were acquired by a single operator.

### Hip-knee-ankle measurement

During the registration process, surgeons defined the planned HKA at a desired knee flexion angle, for both rUKA and mUKA. The postoperative/final HKA was measured using the intraoperative data collected with the optical reference arrays. The femur and tibia mechanical axes were defined from the CT scans. Then, following implantation, the surgeon measured the final HKA with the knee at the same flexion angle as during the planning phase. The postoperative/final HKA was compared to the planned HKA.

### Soft tissue assessment

Following completion of the bone resections the key soft tissues structures (medial collateral ligament [or lateral collateral ligament in a lateral UKA], posterior cruciate ligament, anterior cruciate ligament, patella tendon, and posterior capsule) were inspected to check for iatrogenic damage. Each structure was graded for integrity and function for both rUKA and mUKA groups.

### Statistical analysis

The resection accuracy was calculated by comparing the surgical plan and the eventual measured resection angles and depths. The mean and standard deviation were calculated for the signed and unsigned (absolute) errors. The signed error, defined as *Signed error = Target – Measured*, preserves the direction of the deviation while, the unsigned (absolute) error is the absolute magnitude of the deviation, defined as *Absolute Error = |Target – Measured|* and reflects the size of the deviation regardless of direction. A two one-sided tests (TOST) hierarchical framework was used to compare the resection accuracy between the rUKA and mUKA cohorts. Noninferiority was first assessed using TOST at a significance level of 0.05, with the noninferiority margins of 0.5° and 0.5 mm defined a priori. If noninferiority was demonstrated, superiority was subsequently evaluated using TOST at a significance level of 0.05. To compare the frequency of outliers (cases outside ± 3°) between the 2 cohorts, McNemar’s tests, at a significance level of 0.05, were performed. The threshold of ± 3° was defined based on prior studies [13,14].

Sample size was calculated a priori using previous literature that assessed the resection accuracy of an imageless robotic-assisted device for UKA [13,14]. It was evaluated that a minimum sample size of 22 knees was required per group with a significance level of 0.05 to achieve a power of 80%.

In addition to the accuracy assessments of the full study cohort, assessments of the accuracy of the medial and lateral unicompartmental procedures were assessed independently. Statistical analyses were performed by the study team, which included employees of the system manufacturer.

## Results

**Figure 2:**
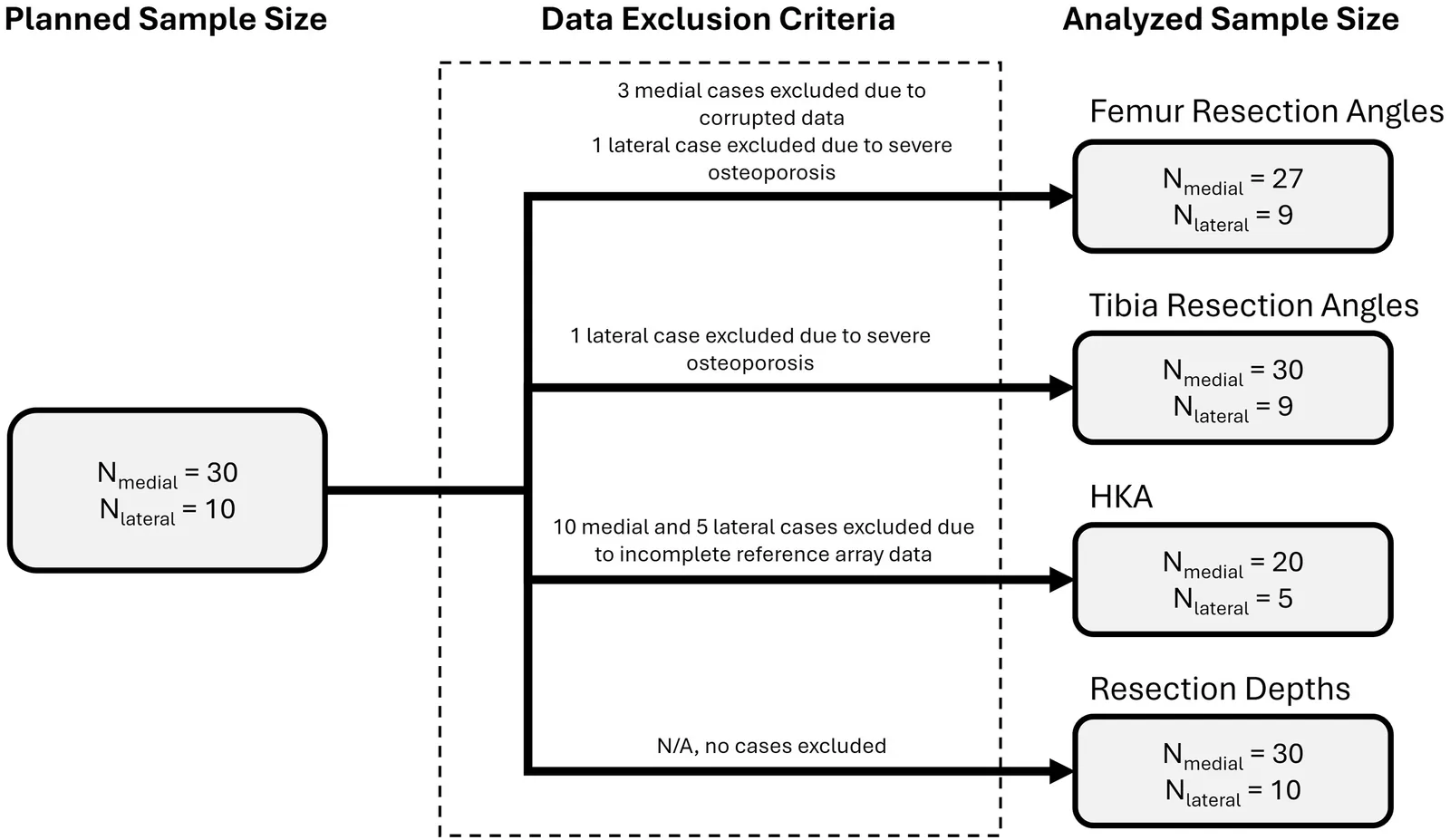
Flow diagram illustrating data exclusion across measurement categories. The total planned cases are shown on the left and the analyzed sample size, after data exclusion, are shown on the right for both medial and lateral cohorts. Where the analyzed sample size differed from the planned sample size, the reason for exclusion is indicated.

In this study, 80 total procedures were completed. Data quality checks identified 3 samples, in the medial UKA cohort, that were excluded from the resection angle measurements due to data corruption. Additionally, 1 specimen in the lateral UKA cohort, exhibited severe osteoporosis, which hindered reliable delineation of the resection planes during the plane-based measurements (Fig. 2). These exclusions were necessary because corrupted files and osteoporotic bone lacked reliable data required to construct bone coordinate systems and plane-based measurements. Reference array tracking data was incomplete for 10 medial UKA and 5 lateral UKA cases when measuring HKA (Fig. 2). Despite the missing data, the remaining sample size was still sufficient to address the primary objectives of the study (Table 2).

**Table 2:** Descriptive statistics of resection accuracy (defined as Error = Target − Measurement) in bilateral cadaveric UKA surgeries separated by compartment (medial, lateral, medial + lateral) and by cohort (rUKA and mUKA).

Measurement	Robotic-assisted			Manual			*P* value	Results of rUKA compared to mUKA
	Sample size	Mean ± SD	Mean Abs ±SD	Sample size	Mean ± SD	Mean Abs ±SD		
Resection angles	Medials and laterals							
Femur V-V	36^a^^b^	0.82 ± 1.92	1.68 ± 1.22	36^a^^b^	−0.22 ± 3.65	2.68 ± 2.44	0.019	Superior
Femur F-E	36^a^^b^	0.54 ± 1.52	1.21 ± 1.06	36^a^^b^	−0.86 ± 5.82	4.54 ± 3.67	<0.001	Superior
Femur I-E	36^a^^b^	0.59 ± 4.61	1.93 ± 4.21	36^a^^b^	0.10 ± 5.05	3.99 ± 3.02	0.010	Superior
Tibia V-V	39^a^	−0.02 ± 1.90	1.57 ± 1.05	39^a^	−0.27 ± 3.7	2.48 ± 1.79	0.006	Superior
Tibia slope	39^a^	0.98 ± 1.18	1.22 ± 0.92	39^a^	0.31 ± 2.93	2.17 ± 1.97	0.005	Superior
HKA	25^c^^d^	0.10 ± 1.44	1.09 ± 0.91	25^c^^d^	0.58 ± 1.60	1.42 ± 0.89	0.001	Noninferior
Resection depths								
Femur P-D	40	0.21 ± 0.73	0.56 ± 0.51	40	0.69 ± 0.57	0.75 ± 0.48	0.050	Superior
Femur A-P	40	0.18 ± 0.86	0.69 ± 0.53	40	−0.33 ± 0.72	0.65 ± 0.45	<0.001	Noninferior
Tibia P-D	40	−1.02 ± 0.59	1.05 ± 0.52	40	−0.28 ± 1.56	1.31 ± 0.87	<0.001	Noninferior
Resection angles	Medials							
Femur V-V	27^b^	1.39 ± 1.76	1.85 ± 1.25	27^b^	1.15 ± 2.58	2.13 ± 1.83	0.045	Noninferior
Femur F-E	27^b^	0.59 ± 1.57	1.18 ± 1.18	27^b^	−0.74 ± 5.58	4.37 ± 3.45	<0.001	Superior
Femur I-E	27^b^	0.94 ± 5.16	1.95 ± 4.86	27^b^	0.46 ± 4.96	4.01 ± 2.84	0.031	Superior
Tibia V-V	30	0.01 ± 1.94	1.62 ± 1.02	30	0.28 ± 3.15	2.51 ± 1.86	0.017	Superior
Tibia slope	30	0.75 ± 1.14	1.06 ± 0.84	30	−0.12 ± 2.94	2.18 ± 1.94	0.003	Superior
HKA	20^c^	0.27 ± 1.37	1.07 ± 0.86	20^c^	0.63 ± 1.70	1.56 ± 0.88	0.030	Superior
Resection depths								
Femur P-D^e^	30	0.17 ± 0.56	0.45 ± 0.37	30	0.59 ± 0.54	0.68 ± 0.42	0.025	Superior
Femur A-P	30	0.17 ± 0.75	0.60 ± 0.47	30	−0.48 ± 0.68	0.68 ± 0.48	<0.001	Noninferior
Tibia P-D	30	−1.09 ± 0.54	1.09 ± 0.53	30	−0.46 ± 1.49	1.30 ± 0.83	<0.001	Noninferior
Resection angles	Laterals							
Femur V-V	9^a^	−0.89 ± 1.29	1.15 ± 1.03	9^a^	−4.33 ± 3.32	4.33 ± 3.32	0.008	Superior
Femur F-E	9^a^	0.39 ± 1.45	1.29 ± 0.64	9^a^	−1.22 ± 6.84	5.05 ± 4.45	0.012	Superior
Femur I-E	9^a^	−0.46 ± 2.16	1.87 ± 0.98	9^a^	−1.00 ± 5.47	3.93 ± 3.70	0.045	Noninferior
Tibia V-V	9^a^	−0.15 ± 1.89	1.39 ± 1.19	9^a^	−2.09 ± 2.02	2.35 ± 1.67	0.039	Noninferior
Tibia slope	9^a^	1.75 ± 1.01	1.75 ± 1.01	9^a^	1.77 ± 2.52	2.13 ± 2.19	0.166	No Stat. Signif.
HKA	5^d^	−0.62 ± 1.66	1.18 ± 1.21	5^d^	0.37 ± 1.20	0.87 ± 0.80	0.402	No Stat. Signif.
Resection depths								
Femur P-D	10	0.32 ± 1.14	0.91 ± 0.7	10	0.97 ± 0.59	0.97 ± 0.59	0.047	Noninferior
Femur A-P	10	0.19 ± 1.17	0.94 ± 0.65	10	0.11 ± 0.68	0.55 ± 0.37	0.343	No Stat. Signif.
Tibia P-D	10	−0.79 ± 0.71	0.93 ± 0.49	10	0.26 ± 1.72	1.35 ± 1.01	0.008	Noninferior

Descriptive statistics include sample size, mean, and standard deviation and their absolute counterparts.

a One case excluded due to severe osteoporosis.

b Three cases excluded due to corrupted data.

c Ten cases excluded due to incomplete reference array data.

d Five cases excluded due to incomplete reference array data.

e Proximal-Distal (P-D)

**Figure 3:**
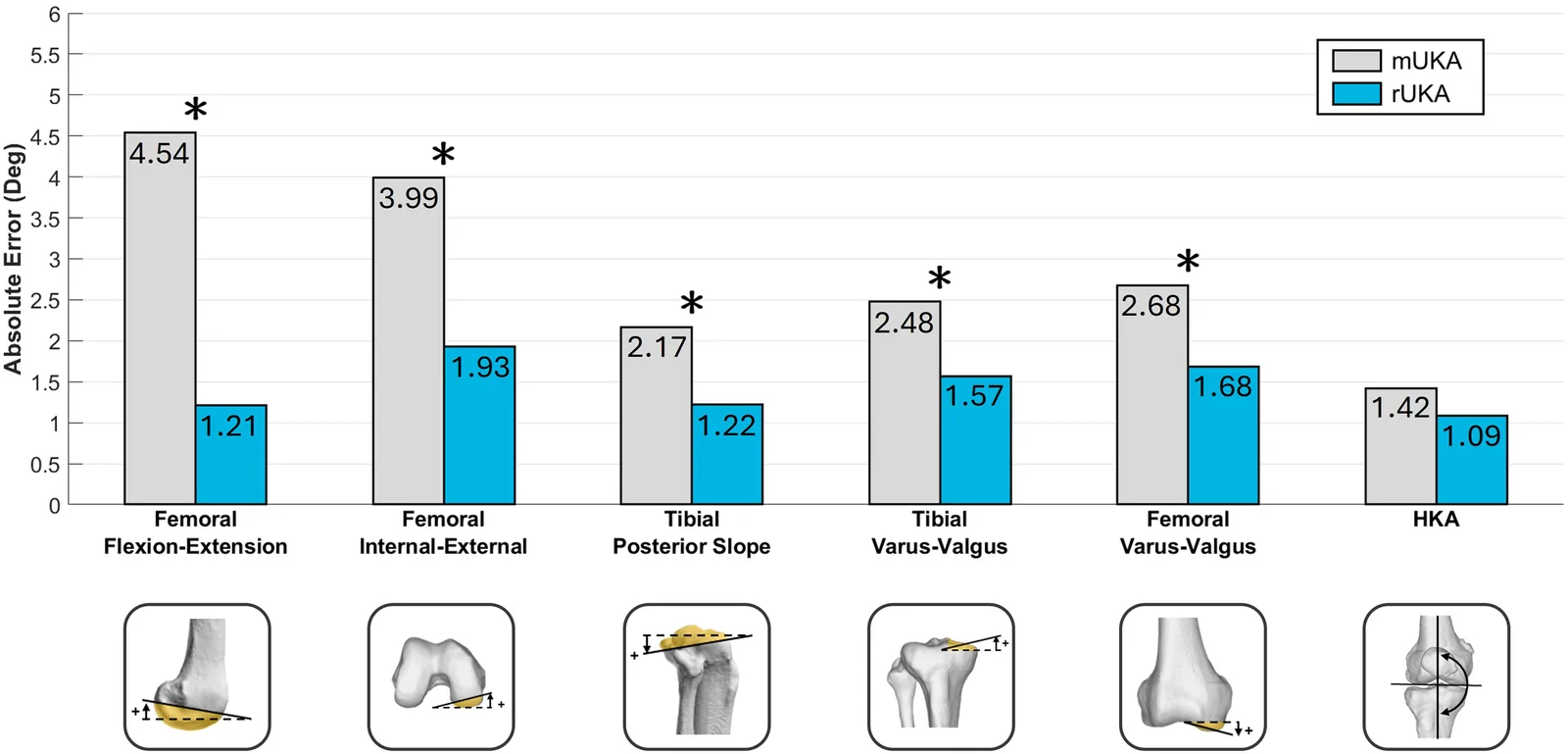
Femur and tibia mean absolute resection angular errors for mUKA and rUKA groups. Error was determined as the mean difference between the intraoperative plan and measured value. An asterisk indicates statistical superiority between the 2 groups (*P* ≤ .05).

The rUKA cohort demonstrated significantly greater alignment accuracy of the femoral component in V-V, F-E, and I-E than mUKA (Fig. 3). The rUKA cohort demonstrated significantly improved alignment accuracy of the tibial component in V-V and slope compared to mUKA (Fig. 3). The accuracy of the HKA was noninferior between the rUKA and mUKA cohorts (mean absolute error 1.09° vs 1.42°, *P* = .001) (Table 2). The accuracy of the resection depths were broadly similar with both rUKA and mUKA delivering mean absolute errors ranging from 0.56 to 0.75 mm on the femur and 1.05 to 1.31 mm on the tibia (Table 2). When analyses were stratified by medial and lateral compartment procedures similar trends were observed with rUKA showing improved component alignment accuracy compared to mUKA; however, due to the reduced sample size in these groups, statistical significance was not reached for all the same metrics (Table 2). Conversely when the HKA analysis was limited to the medial only, the HKA was significantly improved for rUKA compared to mUKA (mean absolute error of 1.07° vs 1.56°, *P* = .03) (Table 2).

**Figure 4:**
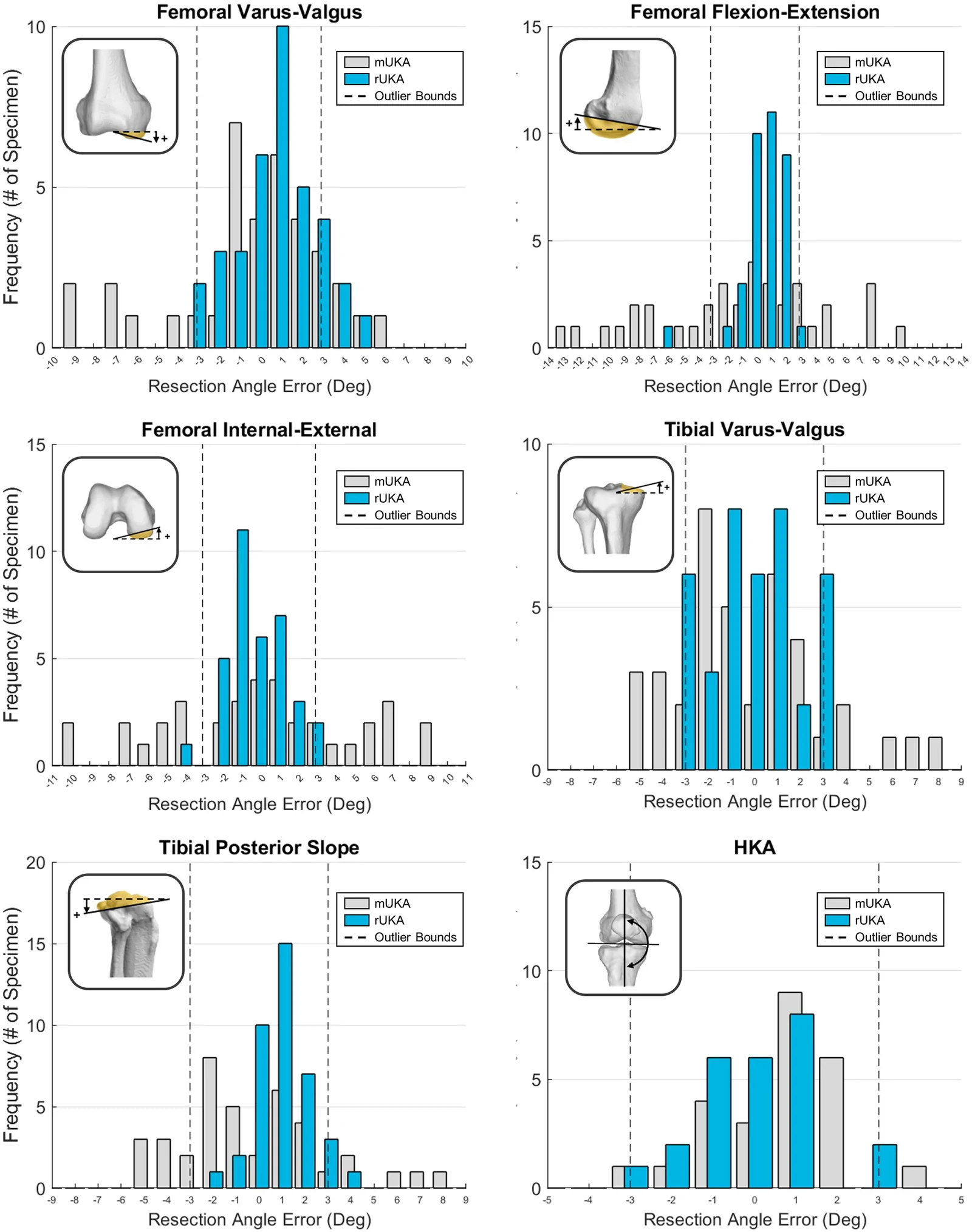
Histograms of femur and tibia resection angle errors for mUKA and rUKA groups. Error was determined as the difference between the intraoperative plan and measured value.

The rUKA results generally had reduced variability than the mUKA with fewer outliers (Fig. 4) with femur F-E, femur I-E, and tibia F-E reaching statistical significance for the number of outliers outside ± 3° (*P* < .05) demonstrating improved precision of the rUKA compared to mUKA. The rUKA data are well centered with the mode error being 0°, 1° or −1° for all angles assessed demonstrating precision and accuracy of the system (Fig. 4).

No intraoperative damage was observed to any of the defined key soft tissue structures in either the rUKA or cUKA cohorts.

## Discussion

The primary finding of this cadaveric study was the use of the assessed rUKA system is associated with improved alignment accuracy compared to the use of manual instrumentation. The results are broadly consistent with the findings of accuracy studies on other image-based and image-free robotic-assisted systems for UKA both in terms of comparisons to manual instrumentation and the magnitude of the root mean square errors reported [9,10,13,15,16].

Improved tibial coronal alignment accuracy is particularly notable considering that varus positioning of tibial components has been associated with an increased risk of revision [7,17]. However, it is currently unknown if the magnitude of improvement in accuracy measured in this study is sufficient to meaningfully influence the clinical survivorship as at present, there is no universally accepted angular threshold that defines a clinically important improvement in UKA component positioning, and much of the available literature relates malposition to revision risk rather than demonstrating a direct survivorship benefit from reducing mean errors. In this context, the clinical relevance of the present findings may be better interpreted in terms of consistency and avoidance of larger deviations, as reflected by the reduction in alignment outliers beyond 3 degrees, rather than by small differences in mean absolute error alone. Further clinical studies with adequate follow-up are required to determine whether the observed reductions in variability and outlier frequency translate into improved patient outcomes or implant survivorship. It is important to note that implant alignment and resection accuracy represent 1 component of the factors that affect clinical outcomes. Additional factors such as weight-bearing limb alignment, soft tissue balance, and hip and ankle anatomy also affect clinical outcomes.

The resection thicknesses were broadly similar between rUKA and manual with both methods delivering a high level of accuracy. This finding is intuitive given that manual instrumentation of Sigma HP incorporates fixed cutting guides with single reference planes to cut slot designs allowing a high level of precision in the resection depth [18]. However, where the rUKA system has a clear advantage is in the orientation of the resections which can be highly dependent on leg positioning and jig orientation with the manual instrumentation. Further, the ability to plan and adjust the orientation of the implants with rUKA can aid in the final implant tracking.

Both rUKA and the manual instrumentation showed a high level of accuracy of the resultant HKA relative to the surgical plan with rUKA significantly improved compared to mUKA when the analysis was restricted to medial procedures. HKA precision is particularly important in UKA as over or undercorrection of the deformity can result in disease progression or loosening, both of which are common reasons for UKA failures [5,6].

To the authors’ knowledge this is the first study that has assessed the accuracy of a robotic-assisted device for both medial and lateral procedures. Despite the fact that lateral UKA procedures represent only 2-3% of UKA [5,6], they have been identified as a highly successful procedure [19,20] with refinements in patient selection, implant design, and surgical technique leading to increased popularity of the procedure [21,22]. Therefore, the data in this study can give confidence that the rUKA system assessed can be accurate in these procedures.

The study found no iatrogenic soft tissue damage in the rUKA cohort and only 1 incident of posterior medial capsule damage in the mUKA cohort, demonstrating that both robotic-assisted and manual instrumentation can be used safely and that the robotic-assisted device can be used without compromising periarticular soft tissue. This is consistent with the findings of prior cadaveric and clinical studies on the same system when used in TKA [14,23] and is pertinent because, while the surgeon has full control of the saw within the resection plane, due to the saw’s connection to the planer articulation and adjustments being made by the system to keep the saw in plane, there may be differences in the surgeon’s proprioception compared to using a saw in a manual procedure. In the mUKA cohort, only 1 incident of iatrogenic soft tissue damage occurred in the posterior medial capsule.

The main limitation of the study was the use of cadaveric specimens which can have variable bone quality potentially impacting both the surgical execution and the measurement repeatability. Further, cadaveric specimens generally have different patterns of bone loss and ligament tension than the normal patient UKA population. A further study limitation was that the accuracy was measured based on the resected bone as compared to the resultant implant position. However, errors introduced during implantation are likely to be equal between the 2 methodologies being assessed maintaining the validity of the findings. Further, in a prior study on TKA that assessed both bone resections and final implant position, a high level of consistency was observed between both sets of data with the implant position being driven by the resection positions [14]. Although the repeatability and reproducibility of the plane-based measurements were not formally assessed, the segmentation and plane-based measurements were conducted by operators blinded to cohort allocation. The plane-fitting approach used is inherently robust to minor operator variation, as each plane is defined using hundreds of points. While operator variability in surface isolation is expected, formal quantification of intraobserver and interobserver reliability would strengthen the technical rigor and should be addressed in future studies, particularly to confirm that measurement variability is small relative to the observed effect sizes. While data exclusions reduced the available sample size relative to the originally planned sample size, and in turn the statistical power of the study, the study was adequately powered to demonstrate the accuracy of all the rUKA procedures compared to manual instrumentation. This means that when subanalyses were performed on the medial and lateral procedures, the resultant analyses were underpowered. Nonetheless, the consistent trends with rUKA resulting in more accurate alignment than mUKA was observed in all 3 analyses (all, medial, and lateral), which can give good confidence that the system performs well compared to manual instrumentation in both use cases.

Additionally, a formal learning curve analysis was not performed. Reported rUKA learning curves primarily relate to operative time/workflow rather than resection accuracy (the primary endpoint here) [16]. This study was conducted prior to clinical release of the UKA application, so all surgeons were early adopters, and the number of cases per surgeon was below the ∼10 to 20 cases over which learning effects are commonly reported. In addition, protocol-driven interruptions for data capture/feedback would have confounded efficiency metrics and limited the interpretability of case-order comparisons.

The use of a match pair study design is advantageous, as comparisons are performed within the same case, ensuring that incomplete cases do not contribute asymmetrically across cohorts. While the match paired analysis strengthens within case comparison and reduces variance, it does not eliminate the possibility that excluded cases may differ systematically from included cases. Future studies should include a sensitivity analysis after data exclusion. Manufacturer involvement (including study execution and analysis) is disclosed in the author affiliations/disclosures; no independent third-party statistical analysis or external validation was performed, and findings should be interpreted accordingly.

A strength of this study is the use of independent methodologies of accuracy assessment, which includes all potential contributions of error including registration error, system errors (camera tracking, saw positioning) and cut execution. Further the results are reported as the absolute error to ensure there is no cancellation of positive and negative data. This is not always the case as, for example, a recent review identifying many studies that did not meet these criteria [24]. The study had a statistically powered sample size and included 6 different surgeons with varying levels of experience with UKA and technology and this further increased the validity and generalizability of the study findings.

## Conclusions

This extensive cadaveric study has demonstrated that the VELYS RAS for UKA has improved alignment accuracy of the femoral and tibial resections compared to manual instrumentation for both medial and lateral procedures. This improved resection accuracy may support more consistent execution of the intraoperative plan and reduced alignment outliers; however, this cadaveric study did not evaluate clinical outcomes, and any impact on patient function or implant survivorship remains to be demonstrated.

## Funding

This study was funded by DePuy Synthes Joint Reconstruction, a Johnson & Johnson Company. The sponsor provided recommendations on study design, analysis, and interpretation of the data, writing of the report, and the decision to submit the article for publication.

## Conflict of interest

David F. Dalury receives royalties from DePuy Synthes; receives Honoria for speakers bureau activities and paid presentations for Vertex; is a paid consultant for DePuy Synthes; holds stock or stock options in PolarisAR; Gary W Doan is a paid employee of DePuy Synthes and holds stock or stock options in DePuy Synthes; Ian J Leslie is a paid employee of DePuy Synthes; Patrick Courtis a paid employee of DePuy Synthes and holds stock or stock options in DePuy Synthes; Ugo Ihekweazu receives Honoria for speakers bureau activities and paid presentations for DePuy; is a paid consultant for DePuy, Ethicon; holds stock or stock options in RevelAi, Visie, RomTech; receives research support from DePuy; and serves as a board member for AAHKS. All other authors declare no potential conflicts of interest.

For full disclosure statements refer to https://doi.org/10.1016/j.artd.2026.102118.

## CRediT authorship contribution statement

**Gary W. Doan:** Writing – review & editing, Writing – original draft, Validation, Methodology, Investigation, Formal analysis, Data curation, Conceptualization. **R. Patrick Courtis:** Writing – review & editing, Supervision, Methodology, Investigation, Data curation, Conceptualization. **Ian J. Leslie:** Writing – review & editing, Writing – original draft, Methodology, Investigation, Data curation, Conceptualization. **Ugo Ihekweazu:** Writing – review & editing, Investigation, Data curation. **David F. Dalury:** Writing – review & editing, Investigation, Data curation.

## Appendix A Supplementary data

Conflict of Interest Statement for Dalury

Conflict of Interest Statement for Doan

Conflict of Interest Statement for Leslie

Conflict of Interest Statement for Courtis

Conflict of Interest Statement for Ihekweazu
