# Supplementary material for: Improved Accuracy With a Novel Imageless Robotic-Assisted System Versus Manual Instrumentation in Unicondylar Knee Arthroplasty: A Cadaveric Study
Source: Arthroplast Today. 2026 Aug 24;41:102118. doi: 10.1016/j.artd.2026.102118 (PMC13528223; doi:10.1016/j.artd.2026.102118)
Supplement: Supplementary file 5 [file mmc5.pdf]

# CONFLICT OF INTEREST STATEMENT

## *American Association of Hip and Knee Surgeons*

(Adopted from the American Academy of Orthopaedic Surgeons disclosure statement)

The following form **must be filled out completely and submitted by each author (example, 6 authors, 6 forms). All items require a response. If there is no relevant disclosure for a given item, enter "*None*."**

Manuscript Title Improved Accuracy with a Novel Imageless Robotic-Assisted System Versus Manual Instrumentation in Unicondylar Knee Arthroplasty: A Cadaveric Study

1. Royalties from a company or supplier (The following conflicts were disclosed)

None

2. Speakers bureau/paid presentations for a company or supplier (The following conflicts were disclosed)

Depuy

3A. Paid employee for a company or supplier (The following conflicts were disclosed)

None

3B. Paid consultant for a company or supplier (The following conflicts were disclosed)

Depuy, Ethicon

3C. Unpaid consultants for a company or supplier (The following conflicts were disclosed)

None

4. Stock or stock options in a company or supplier (The following conflicts were disclosed)

RevelAi, Visie, RomTech

5. Research support from a company or supplier as a Principal Investigator (The following conflicts were disclosed)

Depuy

6. Other financial or material support from a company or supplier (The following conflicts were disclosed)

None

7. Royalties, financial or material support from publishers (The following conflicts were disclosed)

None

8. Medical/Orthopaedic publications editorial/governing board (The following conflicts were disclosed)

None

9. Board member/committee appointments for a society (The following conflicts were disclosed)

AAHKS

**Each author must sign AND print or type his/her name, date and submit a separate form**

In addition, one BLINDED Conflict of Interest form (no author names used) should be submitted per manuscript with all author disclosures.

Ugonna N Ihekweazu

Author Name (Print or Type)

Author Signature

01/27/2026

Date
